# Bis[2-(3-bromo­prop­oxy)-5-methyl­phen­yl]methane

**DOI:** 10.1107/S1600536809010587

**Published:** 2009-03-28

**Authors:** Zahid Hussain, Muhammad Raza Shah, Itrat Anis, Seik Weng Ng

**Affiliations:** a HEJ Research Institute of Chemistry, International Center for Chemical and Biological Sciences, University of Karachi, Karachi 75270, Pakistan; b Department of Chemistry, University of Malaya, 50603 Kuala Lumpur, Malaysia

## Abstract

The title mol­ecule, C_21_H_26_Br_2_O_2_, is a substituted diphenyl­methane derivative whose angle at the methyl­ene carbon is 115.0 (2)°.

## Related literature

For the structure of bis­[2-(3-bromo­prop­oxy)-5-*tert*-butyl­phen­yl]methane, see: Yordanov *et al.* (1995*a* ▶,*b* ▶).
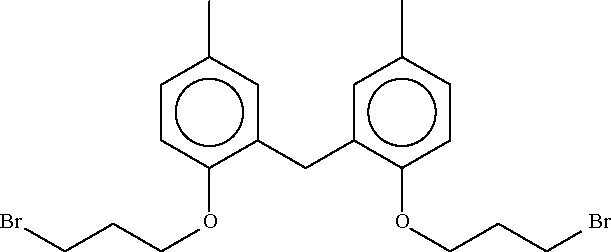


## Experimental

### 

#### Crystal data

C_21_H_26_Br_2_O_2_
*M* *_r_* = 470.24
Monoclinic, 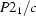

*a* = 12.0054 (2) Å
*b* = 11.9336 (2) Å
*c* = 14.2827 (2) Å
β = 100.777 (1)°
*V* = 2010.16 (6) Å^3^
*Z* = 4
Mo *K*α radiation
μ = 4.04 mm^−1^
*T* = 423 K
0.32 × 0.26 × 0.08 mm

#### Data collection

Bruker SMART APEX diffractometer
Absorption correction: multi-scan (*SADABS*; Sheldrick, 1996 ▶) *T*_min_ = 0.358, *T*_max_ = 0.738
18780 measured reflections
4618 independent reflections
3627 reflections with *I* > 2σ(*I*)
*R*_int_ = 0.046

#### Refinement

*R*[*F* ^2^ > 2σ(*F* ^2^)] = 0.031
*wR*(*F* ^2^) = 0.079
*S* = 1.01
4618 reflections
228 parameters
H-atom parameters constrained
Δρ_max_ = 0.48 e Å^−3^
Δρ_min_ = −0.62 e Å^−3^

### 

Data collection: *APEX2* (Bruker, 2008 ▶); cell refinement: *SAINT* (Bruker, 2008 ▶); data reduction: *SAINT*; program(s) used to solve structure: *SHELXS97* (Sheldrick, 2008 ▶); program(s) used to refine structure: *SHELXL97* (Sheldrick, 2008 ▶); molecular graphics: *X-SEED* (Barbour, 2001 ▶); software used to prepare material for publication: *publCIF* (Westrip, 2009 ▶).

## Supplementary Material

Crystal structure: contains datablocks global, I. DOI: 10.1107/S1600536809010587/tk2401sup1.cif

Structure factors: contains datablocks I. DOI: 10.1107/S1600536809010587/tk2401Isup2.hkl

Additional supplementary materials:  crystallographic information; 3D view; checkCIF report

## Figures and Tables

**Fig. 1.**
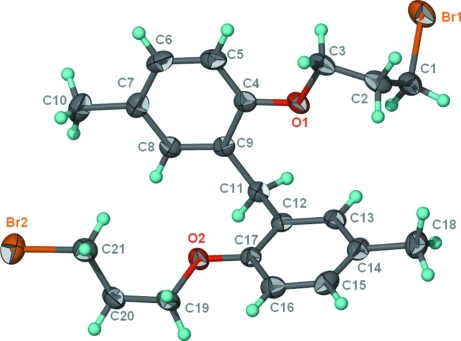
Thermal ellipsoid plot (Barbour, 2001) plot of C21H26O2Br2 at the 70% probability level; hydrogen atoms are drawn as spheres of arbitrary radius.

## supplementary crystallographic information

### Experimental

Potassium carbonate (414 mg, 3 mmol) and 2,2'-methylenebis(4-methylphenol) (228 mg, 1 mmol) in acetone (15 ml) were treated with 1,3-dibromopropane (1.05 g, 5 mmol) at 323 K for 3 h. The solvent was removed under reduced pressure and the residue was dissolved in a mixture of water (50 ml) and dichloromethane (50 ml). The two phases were separated and the aqueous layer extracted with dichloromethane. The dichloromethane solution was dried and the solvent allowed to evaporate slowly to give colorless crystals (182 mg, 80% yield).

### Refinement

Carbon-bound H-atoms were placed in calculated positions (C—H 0.93 to 0.99 Å) and were included in the refinement in the riding model approximation, with *U*(H) = 1.2 to 1.5*U*(C).

### Crystal data

**Table:**

C_21_H_26_Br_2_O_2_	*F*(000) = 952
*M**_r_* = 470.24	*D*_x_ = 1.554 Mg m^−^^3^
Monoclinic, *P*2_1_/*c*	Mo *K*α radiation, λ = 0.71073 Å
Hall symbol: -P 2ybc	Cell parameters from 5910 reflections
*a* = 12.0054 (2) Å	θ = 2.4–28.1°
*b* = 11.9336 (2) Å	µ = 4.04 mm^−^^1^
*c* = 14.2827 (2) Å	*T* = 423 K
β = 100.777 (1)°	Plate, colorless
*V* = 2010.16 (6) Å^3^	0.32 × 0.26 × 0.08 mm
*Z* = 4	

### Data collection

**Table:**

Bruker SMART APEX diffractometer	4618 independent reflections
Radiation source: fine-focus sealed tube	3627 reflections with *I* > 2σ(*I*)
graphite	*R*_int_ = 0.046
ω scans	θ_max_ = 27.5°, θ_min_ = 1.7°
Absorption correction: multi-scan (*SADABS*; Sheldrick, 1996)	*h* = −15→15
*T*_min_ = 0.358, *T*_max_ = 0.738	*k* = −15→15
18780 measured reflections	*l* = −18→18

### Refinement

**Table:**

Refinement on *F*^2^	Primary atom site location: structure-invariant direct methods
Least-squares matrix: full	Secondary atom site location: difference Fourier map
*R*[*F*^2^ > 2σ(*F*^2^)] = 0.031	Hydrogen site location: inferred from neighbouring sites
*wR*(*F*^2^) = 0.079	H-atom parameters constrained
*S* = 1.01	*w* = 1/[σ^2^(*F*_o_^2^) + (0.034*P*)^2^ + 1.182*P*] where *P* = (*F*_o_^2^ + 2*F*_c_^2^)/3
4618 reflections	(Δ/σ)_max_ = 0.001
228 parameters	Δρ_max_ = 0.48 e Å^−^^3^
0 restraints	Δρ_min_ = −0.62 e Å^−^^3^

### Fractional atomic coordinates and isotropic or equivalent isotropic displacement parameters (Å^2^)

**Table:**

	*x*	*y*	*z*	*U*_iso_*/*U*_eq_	
Br1	0.89529 (2)	0.57379 (2)	0.328900 (19)	0.03798 (9)	
Br2	−0.01238 (2)	0.17450 (2)	0.39338 (2)	0.04079 (9)	
O1	0.61771 (14)	0.43666 (12)	0.39928 (11)	0.0255 (3)	
O2	0.38760 (13)	0.14783 (13)	0.48653 (10)	0.0244 (3)	
C1	0.8608 (2)	0.4599 (2)	0.41785 (17)	0.0291 (5)	
H1A	0.8199	0.3970	0.3814	0.035*	
H1B	0.9325	0.4303	0.4552	0.035*	
C2	0.7896 (2)	0.5065 (2)	0.48479 (16)	0.0290 (5)	
H2A	0.7851	0.4499	0.5347	0.035*	
H2B	0.8280	0.5734	0.5167	0.035*	
C3	0.6711 (2)	0.53852 (19)	0.43790 (17)	0.0277 (5)	
H3A	0.6297	0.5714	0.4851	0.033*	
H3B	0.6728	0.5940	0.3866	0.033*	
C4	0.5034 (2)	0.43657 (19)	0.36428 (15)	0.0229 (5)	
C5	0.4379 (2)	0.5323 (2)	0.34459 (16)	0.0283 (5)	
H5	0.4712	0.6042	0.3575	0.034*	
C6	0.3231 (2)	0.5226 (2)	0.30578 (17)	0.0306 (5)	
H6	0.2789	0.5886	0.2918	0.037*	
C7	0.2715 (2)	0.4188 (2)	0.28700 (15)	0.0270 (5)	
C8	0.3394 (2)	0.32366 (19)	0.30820 (15)	0.0226 (5)	
H8	0.3055	0.2519	0.2960	0.027*	
C9	0.45441 (19)	0.32978 (18)	0.34640 (14)	0.0206 (4)	
C10	0.1470 (2)	0.4079 (2)	0.24627 (18)	0.0360 (6)	
H10A	0.1241	0.4675	0.1994	0.054*	
H10B	0.1035	0.4143	0.2977	0.054*	
H10C	0.1323	0.3348	0.2152	0.054*	
C11	0.52599 (19)	0.22530 (18)	0.36524 (14)	0.0208 (4)	
H11A	0.5910	0.2323	0.3320	0.025*	
H11B	0.4801	0.1605	0.3372	0.025*	
C12	0.57131 (19)	0.20078 (17)	0.46953 (14)	0.0198 (4)	
C13	0.6855 (2)	0.21308 (18)	0.50828 (15)	0.0231 (5)	
H13	0.7348	0.2410	0.4688	0.028*	
C14	0.7311 (2)	0.18620 (19)	0.60292 (16)	0.0257 (5)	
C15	0.6577 (2)	0.1448 (2)	0.65809 (16)	0.0281 (5)	
H15	0.6867	0.1251	0.7225	0.034*	
C16	0.5428 (2)	0.1311 (2)	0.62258 (16)	0.0269 (5)	
H16	0.4941	0.1028	0.6623	0.032*	
C17	0.49961 (19)	0.15936 (17)	0.52813 (15)	0.0213 (5)	
C18	0.8558 (2)	0.2004 (2)	0.64178 (18)	0.0346 (6)	
H18A	0.8995	0.1754	0.5941	0.052*	
H18B	0.8771	0.1556	0.6998	0.052*	
H18C	0.8722	0.2796	0.6567	0.052*	
C19	0.3171 (2)	0.0874 (2)	0.53991 (17)	0.0278 (5)	
H19A	0.3126	0.1280	0.5996	0.033*	
H19B	0.3492	0.0121	0.5568	0.033*	
C20	0.2007 (2)	0.07720 (19)	0.47873 (18)	0.0290 (5)	
H20A	0.2074	0.0462	0.4157	0.035*	
H20B	0.1545	0.0246	0.5091	0.035*	
C21	0.1424 (2)	0.1886 (2)	0.46541 (18)	0.0298 (5)	
H21A	0.1860	0.2396	0.4311	0.036*	
H21B	0.1404	0.2219	0.5286	0.036*	

### Atomic displacement parameters (Å^2^)

**Table:**

	*U*^11^	*U*^22^	*U*^33^	*U*^12^	*U*^13^	*U*^23^
Br1	0.03685 (16)	0.04009 (16)	0.04195 (15)	−0.00652 (11)	0.02015 (12)	−0.00002 (11)
Br2	0.03188 (16)	0.04527 (17)	0.04445 (16)	0.00566 (12)	0.00512 (12)	−0.00249 (12)
O1	0.0253 (9)	0.0198 (8)	0.0330 (8)	−0.0047 (6)	0.0098 (7)	−0.0048 (6)
O2	0.0219 (8)	0.0286 (9)	0.0237 (8)	−0.0027 (7)	0.0068 (7)	0.0033 (6)
C1	0.0291 (13)	0.0257 (12)	0.0311 (12)	−0.0052 (10)	0.0018 (10)	−0.0029 (10)
C2	0.0379 (14)	0.0246 (13)	0.0247 (11)	−0.0104 (10)	0.0060 (10)	−0.0057 (9)
C3	0.0350 (14)	0.0217 (12)	0.0297 (12)	−0.0072 (10)	0.0149 (10)	−0.0062 (9)
C4	0.0262 (12)	0.0245 (12)	0.0207 (10)	0.0005 (9)	0.0111 (9)	0.0017 (9)
C5	0.0365 (14)	0.0224 (12)	0.0296 (12)	0.0019 (10)	0.0152 (10)	0.0022 (9)
C6	0.0384 (15)	0.0274 (13)	0.0290 (12)	0.0102 (11)	0.0141 (11)	0.0075 (10)
C7	0.0273 (13)	0.0347 (13)	0.0203 (10)	0.0050 (10)	0.0080 (9)	0.0023 (9)
C8	0.0250 (12)	0.0248 (11)	0.0191 (10)	−0.0009 (9)	0.0067 (9)	0.0006 (9)
C9	0.0251 (12)	0.0225 (11)	0.0156 (9)	0.0013 (9)	0.0079 (8)	0.0013 (8)
C10	0.0287 (14)	0.0459 (16)	0.0325 (13)	0.0088 (11)	0.0033 (11)	0.0040 (11)
C11	0.0239 (12)	0.0205 (11)	0.0191 (10)	−0.0006 (9)	0.0065 (9)	−0.0022 (8)
C12	0.0242 (12)	0.0151 (10)	0.0203 (10)	0.0019 (8)	0.0050 (9)	−0.0028 (8)
C13	0.0258 (12)	0.0177 (11)	0.0264 (11)	−0.0024 (9)	0.0061 (9)	−0.0017 (9)
C14	0.0265 (13)	0.0204 (11)	0.0278 (11)	0.0004 (9)	−0.0009 (9)	−0.0054 (9)
C15	0.0332 (14)	0.0296 (13)	0.0189 (10)	0.0048 (10)	−0.0015 (10)	0.0001 (9)
C16	0.0320 (14)	0.0266 (12)	0.0238 (11)	0.0012 (10)	0.0097 (10)	0.0019 (9)
C17	0.0227 (12)	0.0183 (11)	0.0227 (10)	0.0008 (9)	0.0042 (9)	−0.0014 (8)
C18	0.0270 (14)	0.0361 (15)	0.0370 (13)	−0.0005 (11)	−0.0037 (11)	−0.0039 (11)
C19	0.0281 (13)	0.0241 (12)	0.0338 (12)	−0.0028 (10)	0.0125 (10)	0.0036 (10)
C20	0.0259 (13)	0.0224 (12)	0.0412 (13)	−0.0036 (10)	0.0124 (10)	−0.0024 (10)
C21	0.0314 (14)	0.0244 (12)	0.0351 (12)	−0.0001 (10)	0.0096 (11)	−0.0038 (10)

### Geometric parameters (Å, °)

**Table:**

Br1—C1	1.957 (2)	C10—H10B	0.9800
Br2—C21	1.956 (3)	C10—H10C	0.9800
O1—C4	1.369 (3)	C11—C12	1.516 (3)
O1—C3	1.436 (3)	C11—H11A	0.9900
O2—C17	1.372 (3)	C11—H11B	0.9900
O2—C19	1.435 (3)	C12—C13	1.387 (3)
C1—C2	1.503 (3)	C12—C17	1.398 (3)
C1—H1A	0.9900	C13—C14	1.398 (3)
C1—H1B	0.9900	C13—H13	0.9500
C2—C3	1.503 (4)	C14—C15	1.379 (3)
C2—H2A	0.9900	C14—C18	1.506 (3)
C2—H2B	0.9900	C15—C16	1.388 (3)
C3—H3A	0.9900	C15—H15	0.9500
C3—H3B	0.9900	C16—C17	1.393 (3)
C4—C5	1.386 (3)	C16—H16	0.9500
C4—C9	1.407 (3)	C18—H18A	0.9800
C5—C6	1.391 (4)	C18—H18B	0.9800
C5—H5	0.9500	C18—H18C	0.9800
C6—C7	1.389 (4)	C19—C20	1.509 (3)
C6—H6	0.9500	C19—H19A	0.9900
C7—C8	1.397 (3)	C19—H19B	0.9900
C7—C10	1.505 (3)	C20—C21	1.497 (3)
C8—C9	1.388 (3)	C20—H20A	0.9900
C8—H8	0.9500	C20—H20B	0.9900
C9—C11	1.510 (3)	C21—H21A	0.9900
C10—H10A	0.9800	C21—H21B	0.9900
			
C4—O1—C3	119.08 (18)	C12—C11—H11A	108.5
C17—O2—C19	116.61 (17)	C9—C11—H11B	108.5
C2—C1—Br1	111.78 (17)	C12—C11—H11B	108.5
C2—C1—H1A	109.3	H11A—C11—H11B	107.5
Br1—C1—H1A	109.3	C13—C12—C17	118.14 (19)
C2—C1—H1B	109.3	C13—C12—C11	121.2 (2)
Br1—C1—H1B	109.3	C17—C12—C11	120.6 (2)
H1A—C1—H1B	107.9	C12—C13—C14	122.8 (2)
C3—C2—C1	114.44 (19)	C12—C13—H13	118.6
C3—C2—H2A	108.6	C14—C13—H13	118.6
C1—C2—H2A	108.6	C15—C14—C13	117.3 (2)
C3—C2—H2B	108.6	C15—C14—C18	121.8 (2)
C1—C2—H2B	108.6	C13—C14—C18	120.9 (2)
H2A—C2—H2B	107.6	C14—C15—C16	122.1 (2)
O1—C3—C2	105.84 (19)	C14—C15—H15	118.9
O1—C3—H3A	110.6	C16—C15—H15	118.9
C2—C3—H3A	110.6	C15—C16—C17	119.3 (2)
O1—C3—H3B	110.6	C15—C16—H16	120.3
C2—C3—H3B	110.6	C17—C16—H16	120.3
H3A—C3—H3B	108.7	O2—C17—C16	123.5 (2)
O1—C4—C5	124.4 (2)	O2—C17—C12	116.07 (18)
O1—C4—C9	115.06 (19)	C16—C17—C12	120.4 (2)
C5—C4—C9	120.5 (2)	C14—C18—H18A	109.5
C4—C5—C6	119.7 (2)	C14—C18—H18B	109.5
C4—C5—H5	120.2	H18A—C18—H18B	109.5
C6—C5—H5	120.2	C14—C18—H18C	109.5
C7—C6—C5	121.6 (2)	H18A—C18—H18C	109.5
C7—C6—H6	119.2	H18B—C18—H18C	109.5
C5—C6—H6	119.2	O2—C19—C20	107.90 (19)
C6—C7—C8	117.5 (2)	O2—C19—H19A	110.1
C6—C7—C10	121.8 (2)	C20—C19—H19A	110.1
C8—C7—C10	120.7 (2)	O2—C19—H19B	110.1
C9—C8—C7	122.7 (2)	C20—C19—H19B	110.1
C9—C8—H8	118.7	H19A—C19—H19B	108.4
C7—C8—H8	118.7	C21—C20—C19	111.2 (2)
C8—C9—C4	118.1 (2)	C21—C20—H20A	109.4
C8—C9—C11	121.2 (2)	C19—C20—H20A	109.4
C4—C9—C11	120.7 (2)	C21—C20—H20B	109.4
C7—C10—H10A	109.5	C19—C20—H20B	109.4
C7—C10—H10B	109.5	H20A—C20—H20B	108.0
H10A—C10—H10B	109.5	C20—C21—Br2	111.45 (17)
C7—C10—H10C	109.5	C20—C21—H21A	109.3
H10A—C10—H10C	109.5	Br2—C21—H21A	109.3
H10B—C10—H10C	109.5	C20—C21—H21B	109.3
C9—C11—C12	114.98 (17)	Br2—C21—H21B	109.3
C9—C11—H11A	108.5	H21A—C21—H21B	108.0
			
Br1—C1—C2—C3	67.6 (2)	C9—C11—C12—C13	−109.0 (2)
C4—O1—C3—C2	170.44 (18)	C9—C11—C12—C17	74.4 (3)
C1—C2—C3—O1	62.5 (2)	C17—C12—C13—C14	−0.2 (3)
C3—O1—C4—C5	14.2 (3)	C11—C12—C13—C14	−176.9 (2)
C3—O1—C4—C9	−167.37 (18)	C12—C13—C14—C15	0.7 (3)
O1—C4—C5—C6	177.6 (2)	C12—C13—C14—C18	179.7 (2)
C9—C4—C5—C6	−0.8 (3)	C13—C14—C15—C16	−0.7 (3)
C4—C5—C6—C7	0.7 (3)	C18—C14—C15—C16	−179.8 (2)
C5—C6—C7—C8	−0.3 (3)	C14—C15—C16—C17	0.3 (4)
C5—C6—C7—C10	179.2 (2)	C19—O2—C17—C16	−10.0 (3)
C6—C7—C8—C9	−0.2 (3)	C19—O2—C17—C12	169.29 (19)
C10—C7—C8—C9	−179.6 (2)	C15—C16—C17—O2	179.4 (2)
C7—C8—C9—C4	0.1 (3)	C15—C16—C17—C12	0.2 (3)
C7—C8—C9—C11	−177.87 (19)	C13—C12—C17—O2	−179.52 (18)
O1—C4—C9—C8	−178.16 (18)	C11—C12—C17—O2	−2.8 (3)
C5—C4—C9—C8	0.4 (3)	C13—C12—C17—C16	−0.2 (3)
O1—C4—C9—C11	−0.2 (3)	C11—C12—C17—C16	176.5 (2)
C5—C4—C9—C11	178.35 (19)	C17—O2—C19—C20	−175.85 (18)
C8—C9—C11—C12	−113.2 (2)	O2—C19—C20—C21	−69.0 (3)
C4—C9—C11—C12	68.9 (3)	C19—C20—C21—Br2	−176.17 (16)
